# Landscape Pattern Evolution Processes of Wetlands and Their Driving Factors in the Xiong’an New Area of China

**DOI:** 10.3390/ijerph18094403

**Published:** 2021-04-21

**Authors:** Miao Yang, Jiaguo Gong, Yong Zhao, Hao Wang, Cuiping Zhao, Qin Yang, Yingshen Yin, Ying Wang, Bo Tian

**Affiliations:** 1 State Key Laboratory of Simulation and Regulation of Water Cycle in River Basin, China Institute of Water Resources and Hydropower Research, Beijing 100038, China; myang0206@126.com (M.Y.); zhaoyong@iwhr.com (Y.Z.); wanghao@iwhr.com (H.W.); zhaocuiping139@163.com (C.Z.); yangqin1214@126.com (Q.Y.); yingwang24@126.com (Y.W.); 2 College of Hydrology and Water Resources, Hohai University, Nanjing 210098, China; 3 College of Water Science and Engineering, Zhengzhou University, Zhengzhou 450001, China; yingshenyin@163.com (Y.Y.); 18722113509@163.com (B.T.)

**Keywords:** Xiong’an New Area, Baiyangdian, landscape pattern, wetland, driving factor

## Abstract

Wetland landscape patterns are the result of various ecological and hydrological processes. Based on the land use landscape types from 1980 to 2017, a transfer matrix, landscape pattern analysis index, and principal component analysis were used to analyze the landscape pattern evolution in the Xiong’an New Area of China, which has a large area with a lake and river wetlands. The results showed that the wetland area has changed greatly since 2000 and the beach land has decreased greatly, while the area of the lake and river wetlands has increased slightly. Beach land was the dominant landscape type of the wetland. The dominant degree of the wetland landscape showed a slightly decreasing trend, and the patches tended to be scattered. The shape complexity of the ponds was the lowest, while that of rivers was the highest. The fragmentation degree of the wetland patches increased, the proportion of landscape types tended to be equalized, and the landscape heterogeneity increased. The leading factors of the wetland landscape change can be summarized as socioeconomic, meteorological, and hydrological processes, with a cumulative contribution rate of 85.3%, among which socioeconomic development was the most important factor. The results have important guiding significance for the ecological restoration and management of wetlands in the Xiong’an New Area and other wetland ecosystems with rivers and lakes.

## 1. Introduction

An important environmental resource, wetlands, are among the most biodiverse ecological landscapes in nature. Wetlands also offer a variety of ecological services such as regulating runoff, improving water quality, regulating microclimate, providing food, and providing tourism resources [1,2]. Some studies have shown that each year, various types of ecosystems worldwide provide services worth at least 33 trillion U.S. dollars including 4.9 trillion U.S. dollars from wetlands [3]. However, the area, number, type, and structure of wetlands have changed significantly in recent decades due to biophysical factors and human activities [4,5]. It is estimated that more than 50% of the world’s wetlands may have been altered, degraded, or lost through a wide range of human activities over the last 150 years [6]; therefore, the protection and rational exploitation of wetlands have become important issues for global ecosystem studies. Since the 1980s, the principles and methods of landscape ecology patterns, processes, scale, and driving mechanism analysis have been gradually applied in the field of wetland science, which has promoted research on the evolution of wetland landscape patterns [7].

The landscape spatial pattern refers to the spatial arrangement of landscape patches of different sizes and shapes, which is an important manifestation of the landscape heterogeneity. Ecological processes are actually the reasons for spatial changes in wetland landscapes while these can influence ecological processes [8,9]. The study of the dynamic change characteristics and evolution trends of wetland landscape patterns is conducive to the protection, restoration, planning, and management of wetland landscapes, and it is an important basis for the construction of regional ecological security patterns. To date, several studies have focused on employing remote sensing (RS) and geographic information system (GIS) technologies to calculate quantitative indicators and analyze the characteristics of landscape patterns at different scales [10,11,12,13,14,15]. Wetland landscape pattern evolution research methods have primarily included qualitative description, landscape ecological chart overlays, and landscape pattern quantitative analysis [16]. The landscape pattern index had been widely adopted to quantitatively describe spatial features of landscape patterns at the patch, class, and landscape scales [17,18]. In addition, the driving mechanism of changes in wetland landscapes and spatiotemporal variations in human influence have increasingly attracted attention [19,20,21,22]. From what has been discussed above, most researchers have focused on wetland landscape pattern changes in the islands, rivers deltas, plains, and plateaus. However, litter information is available on changes in wetland ecosystems with river and lake couplings.

The Xiong’an New Area is a state-level new area established in China in 2017. The protection and restoration of wetlands in this area is an important aspect of guaranteeing ecological security. The wetland resources in the Xiong’an New Area are primarily composed of Baiyangdian Lake and several rivers, which constitute an important buffer for ecological security for the entire north region of China. Although Xiong’an New Area is rich in ecological resources, its ecological environment is also relatively sensitive and fragile. Due to climate change and strong human activities, it is confronted with ecological and environmental problems such as a reduction in wetland area, the aggravation of fragmentation, and a reduction in biodiversity. Currently, researchers have conducted many studies regarding the landscape pattern of Baiyangdian Lake. Some authors have directly analyzed the land cover change and tried to reveal the interaction between land cover change and human activities [23,24]. Others have analyzed the dynamic changes in the landscape pattern of wetland types, and reported the influence of climate, hydrology, economy, society, and policy on the landscape pattern [25,26,27,28,29,30,31,32]. As for the analysis of driving factors of landscape patterns, Bai and Yan et al. [26,27] concluded that atmospheric precipitation was the primary influencing factor of natural wetland change in Baiyangdian Lake. Zhang et al. [25] and Zhuang et al. [28] concluded that population, social and economic development, and water level change were the primary factors affecting the landscape pattern changes in Baiyangdian Lake. Due to the differences in research methods and data, the outcomes of different studies are not identical, and further studies are required. In addition, there is still a lack of research on river wetlands and lake wetlands within the entire Xiong’an New Area.

The objective of this study was to investigate and analyze the dynamic changes in wetland landscape patterns in the Xiong’an New Area of China from 1980 to 2017. For such purposes, based on multi-phase land use data, the impact of grain size on the landscape pattern analysis was considered, and the landscape scale, structure, and pattern index were analyzed. The driving mechanism of the landscape pattern change were also analyzed from the aspects of the meteorological and socio-economic factors and hydrological processes. In addition, the contribution rates of the primary influencing factors were obtained. These results are of great significance to the realization of the goal of building the Xiong’an New Area into a green, low-carbon, information-smart, ecology-led international city, and can provide a reference for the planning and management of wetland ecosystems with river and lake couplings.

## 2. Materials and Methods

### 2.1. Study Area

**Figure 1:**
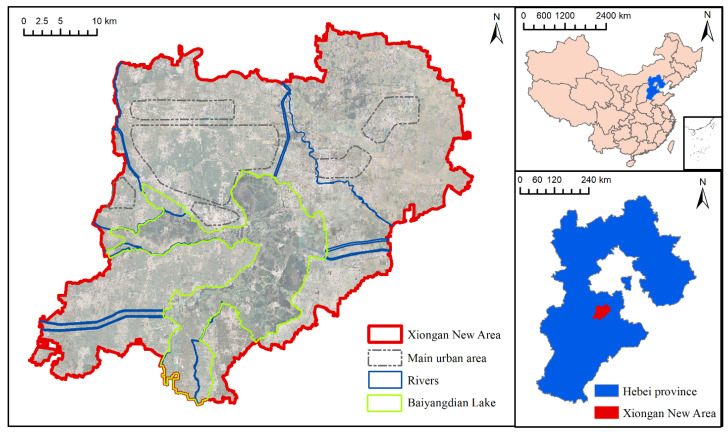
Location of the study area. The main urban area represents the area of the Xiong’an New Area that is planned for future urban development, where a large number of urban water systems and wetlands will be built.

The study area is located in the mid-latitude zone and has a warm temperate monsoon continental climate with four distinct seasons (Figure 1). The average annual temperature is 11.7 °C, the highest average monthly temperature is 26 °C, and the lowest average monthly temperature is −4.9 °C. The average annual rainfall is 551.5 mm, of which 80% occurs from June to September. The frost-free period lasts approximately 185 days (https://baike.so.com/doc/25631265-26683118.html#refff_25631265-26683118-1 (accessed on 20 December 2020)). The entire territory is higher in the northwest and slightly lower in the southeast, ranging from 7–19 m above sea level, approximately 1/1000 of the natural longitudinal slope. The Xiong’an New Area is rich in wetland resources including the entire Baiyangdian Lake and several rivers. Baiyangdian Lake is the largest freshwater lake in north China, and its rivers include the Zhulong River, the Xiaoyi River, the Tang River, the Fu River, the Cao River, the Ping River, the Bao River, and the Baigou Diversion River. The entire wetland system provides enormous ecological benefits with regard to aquatic product supply, climate regulation, flood storage, and biodiversity protection, which is also of great significance for the protection of the ecological security pattern in north China [33].

### 2.2. Data Sources and Processing

The land use data were interpreted using Landsat medium-resolution remote sensing images (http://glovis.usgs.gov/ (accessed on 15 October 2020)) with a resolution of 30 m. The economic and social data of the Xiong’an New Area were obtained from the Baoding City Economic Statistics Yearbook (https://data.cnki.net/area/Yearbook/Single/N2017120334?z=D03 (accessed on 20 November 2020)). They primarily included the total population, urban population, rural population, gross domestic product (GDP), per capita GDP, gross product of the primary industry, gross product of the secondary industry, and gross product of the tertiary industry. Meteorological data and hydrological data were obtained from China’s meteorological science data sharing network (http://data.cma.cn/ (accessed on 10 October 2020)) and from the China Annual Hydrological Report published by the Ministry of Water Resources, Herbei Province Water Resources Bulletin and other relevant sources [34,35,36,37,38,39,40]. These data primarily included precipitation, temperature, the underground water levels of Baoding City, the water levels of Baiyangdian Lake, and the ecologically restored water of Baiyangdian Lake.

### 2.3. Research Methods

#### 2.3.1. Remote Sensing Image Interpretation and Landscape Classification

Land use data products use Landsat remote sensing images as the primary data source. To ensure the accuracy of the interpretation results, the effects of plant growth and seasonal water level changes were comprehensively considered, and the time of the remote sensing image data was primarily from June to August of 1980–2017. After image fusion, geometric correction, image enhancement, and mosaic processing, the remote sensing image was combined with the image supervision classification of the results and other auxiliary data using a full digital human–computer interaction operation method that unified the interpretation of signs. Under guidance, and according to the understanding of the image spectrum, texture, tone, and other factors, these were combined with the terrain visual interpretation. Due to the Landsat 7 scan line error and cloud, the related plug-in of ENVI software was utilized to deal with these factors, and this primarily consisted of masking to repair it using the interpolation method. The data were checked and corrected by means of a random distribution of checkpoints, and the average classification accuracy of each data was greater than 90%.

Land use data of the Xiong’an New Area for 1980, 1990, 2000, 2010, and 2017 were used for the landscape types, and the wetland landscape and non-wetland landscape types were divided into major and minor types (Table 1). The wetlands types were divided according to the Convention on Wetlands of International Importance Especially as Waterfowl Habitat (http://portal.unesco.org/en/ev.phpURL_ID=15398&URL_DO_TOPI-C&URL_SECTION=201.html (accessed on 6 October 2020)), which includes four types of land uses: rivers, lakes, ponds, and beach land. Paddy fields were primarily formed by artificial means during the late 1990s, and were treated as cultivated land resources.

#### 2.3.2. Landscape Conversion Matrix

The landscape conversion matrix can quantitatively show the transformation of different landscape types and has been widely adapted to study land use changes. Generally, ArcGIS software can be used to conduct the conversion matrix analysis on two-phase land use data. This study was primarily divided into three periods: from 1980 to 2000, from 2000 to 2017, and from 1980 to 2017.The conversion matrix can be expressed as follows:

$$A_{ij}=\left|\begin{array}{cc} \begin{array}{cc} A_{11} & A_{12}\\ A_{21} & A_{22} \end{array} & \begin{array}{cc} \ldots & A_{1n}\\ \ldots & A_{11} \end{array}\\ \begin{array}{cc} \ldots & \ldots \\ A_{11} & A_{11} \end{array} & \begin{array}{cc} \ldots & \ldots \\ \ldots & A_{11} \end{array} \end{array}\right|$$

where *a* is area; *n* is the number of landscape types; *i* and *j* are the landscape types in the initial and last study stages, respectively; the rows represent the landscape in the *t* stage; the columns represent the landscape in the *j* in *t* + 1 stage; and *A_ij_* represents the landscape conversion matrix from *t* to *t* + 1.

#### 2.3.3. Landscape Pattern Index

The landscape pattern index can compress the information of the landscape pattern and is a quantitative indicator used to measure the structural composition and characteristic in space. Fragstats 4.2 was used to calculate largest patch index (LPI), mean patch area (MPS), area weighted mean fractal dimension (FRAC_AM), patch cohesion index (COHESION), number of patches (NP), patch density (PD), perimeter area fractal dimension (PAFRAC), aggregation index (AI), and Shannon’s diversity index (SHDI). These were selected at the patch class and landscape level [41,42]. The expressions and ecological meaning are shown in Table 2.

#### 2.3.4. Principal Component Analysis

A principal component analysis (PCA) is a multivariate statistical analysis method that simplifies the data structure by reducing the dimension, and selects the primary variables using a linear transformation from the previously intricate multivariable. PCA attempts to recombine the original indexes with correlations into a new set of interrelated indexes instead of the original indexes. The most classic approach is to select the first linear combination, namely the variance of the first composite index to express, that is, the greater the variance, the more information the first principal component contains. If the first principal component is insufficient to express the original index information, then the second principal component is considered. The number of principal components is determined according to a variance contribution rate greater than 85%.

Using SPSS software, the PCA was performed on the selected indicators to explore the driving force of the wetland evolution in the Xiong’an New Area. As a result of the comprehensive effects of natural and human activities during the evolution of the wetland landscape, 14 relevant driving factors were selected for the PCA, and data from 1980 to 2017 were selected as the analysis sample. To facilitate the data display, X_1_, X_2_, …, X_14_ represented the 14 factors, namely, X_1_: precipitation (mm); X_2_: temperature (°C); X_3_: Baoding city groundwater level (m); X_4_: Baiyangdian water level (m); X_5_: Intake water volume (10^8^ m^3^); X_6_: ecological replenishment volume (10^8^ m^3^); X_7_: Xiong’an gross production value (10^4^ yuan); X_8_: gross product of primary industry (10^4^ yuan); X_9_: gross product of secondary industry (10^4^ yuan); X_10_: gross product of tertiary industry (10^4^ yuan); X_11_: GDP per capita (10^4^ yuan); X_12_: total population (10^4^); X_13_: urban population (10^4^); and X_14_: rural population (10^4^).

## 3. Results and Discussion

### 3.1. Analysis of the Scale and Structural Changes

#### 3.1.1. Landscape Scale Changes

**Figure 2:**
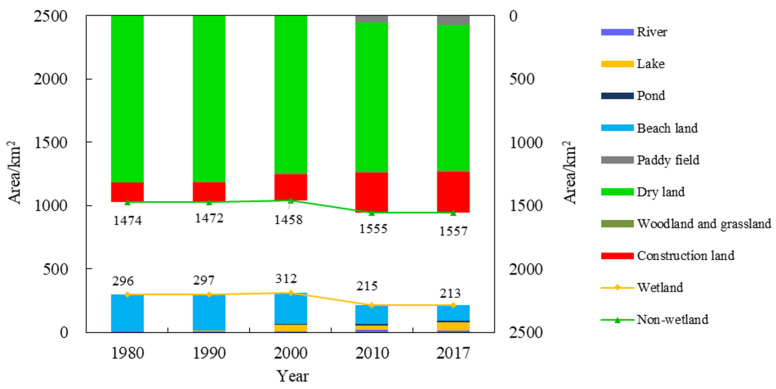
Changes in the area of the landscape types in the Xiong’an New Area from 1980 to 2017.

**Figure 3:**
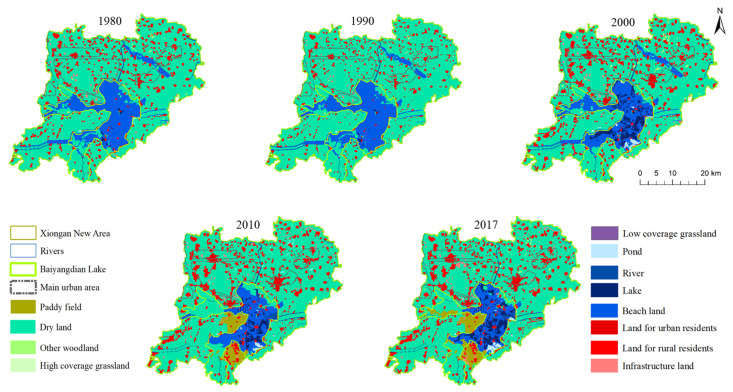
Spatial distribution of the landscape types in the Xiong’an New Area from 1980 to 2017.

The impact of natural factors and human activities on landscape are initially experienced in a change in the landscape scale. The following analysis was conducted from the area change in the wetland and non-wetland types. The upper and lower parts of Figure 2 show the area change trend of the non-wetland and wetland types, respectively. It can be seen that the area of the non-wetland type increased significantly after 2000, while the corresponding wetland area decreased. The increase in the non-wetland area was primarily due to the increase in the area of construction land, and a certain decrease in the area of dry land. However, the area of the paddy field increased. The increase in construction land was primarily related to local economic and social development. The growth in the economy and population causes land types for factories and infrastructure construction to increase. The decrease in dry land was due to the encroachment of construction land and the higher economic benefits of paddy land. Hence, local farmers have transformed dry land into paddy land. As can be seen from Figure 3, after 2000, construction land began to gather mainly in the north of the Xiong’an New Area, and large landscape patches of construction land appeared. Paddy fields were primarily converted from dry land in the west and south of the Baiyangdian Lake margin. This area is low lying and close to Baiyangdian Lake, making it easier to obtain water. According to the results in Figure 2, the area of wetland decreased after 2000; this primarily reflected a reduction in the beach area, and the areas of lakes, ponds, and river wetlands increased to a certain extent. However, this increase was not enough to make up for the reduced area of beach wetland. This may be due to increased water withdrawals by the population and a decrease in natural runoff, which will be further analyzed below. Figure 3 shows that the area of wetland decreased after 2000 and was primarily distributed in the northwest area of Baiyangdian Lake.

**Figure 4:**
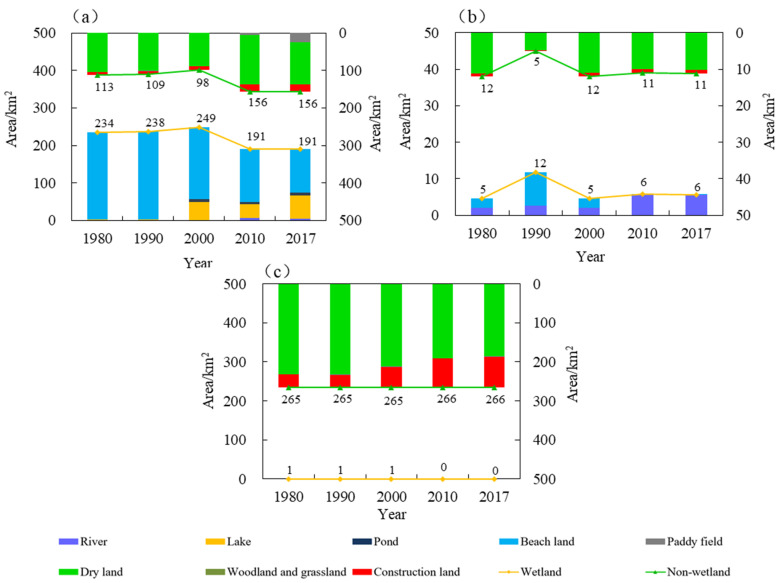
Changes in the area of the different landscape types in the Xiong’an New Area from 1980 to 2017. (**a**) Baiyangdian Lake, (**b**) rivers, and (**c**) main urban area.

In the future, lake wetlands, river wetlands, and urban wetlands will be important components of the wetland ecosystem in the Xiong’an New Area. Figure 4a–c shows the changes in the landscape area within Baiyangdian Lake, rivers, and main urban area, respectively. In terms of wetland area, Baiyangdian Lake had the largest wetland area, followed by the river wetland, and the urban wetland area was the smallest. As can be seen from Figure 4a, Baiyangdian Lake is primarily composed of beaches, lakes, and other wetlands. After 2000, the wetland area decreased significantly. The wetland area ratio in Baiyangdian Lake decreased from 71.75% in 2000 to 55.04% in 2017. The degraded wetlands were reclaimed as dry land, which improved the ratio of non-wetland area. As can be seen from Figure 4b, most of the river wetland areas were dry land, and this was primarily due to a decrease in the amount of water entering the lake. Only the Fu River, Xiaoyi River, and Baigou Diversion River among the eight rivers in Baiyangdian have water all year round, the rest of the rivers are intermittently dry. In addition, the river beaches are gradually being reclaimed into dry land. As can be seen from Figure 4c, the area of urban wetlands has decreased and consists mostly of artificial wetlands or ponds. Currently, the larger constructed wetlands in the Xiong’an New Area are the Anxin County Wetland Park and the Xiong County Hot Spring Lake Park. The artificial wetlands in the Xiong’an New Area still require a great deal of construction to meet the needs of future urban construction and residents’ needs for wetland ecosystem services.

#### 3.1.2. Landscape Structural Change

Generally speaking, there are two types of transformation relationships between wetland landscapes and non-wetland landscapes: gradual change and conversion. The analysis results in Table S1 in the Supplementary Materials show that during 1980–2000, the conversion area from non-wetland to wetland was 18.02 km^2^, with a conversion rate of 1.22%. This primarily manifested as the conversion of dry land to lakes, beach land, and land for residents. The conversion area from wetland to non-wetland was 2.27 km^2^, and the conversion rate was 0.77%, which primarily manifested in the conversion of beach land to dry land and land for rural residents. Conversion in the internal wetland system primarily manifested in the conversion of beach land to lakes and ponds.

It can be seen from the analysis results in Table S2 in the Supplementary Materials that during 2000–2017, the landscape transition was more complicated. The conversion area from non-wetland to wetland was 21.45 km^2^ and the conversion rate was 1.47%. This primarily manifested in the conversion of dry land to rivers, ponds, and beach land, and the conversion of land for rural residents to beach land. The conversion area from wetland to non-wetland was 120.48 km^2^ and the conversion rate was 38.64%. This was primarily manifested in the conversion of beach land to paddy fields, dry land, and land for rural residents. Conversion in the internal wetland system was primarily manifested in the conversion of beach land to rivers, lakes, and ponds. A total of 95.32 km^2^ of beach land was converted to cultivated land, 12.83 km^2^ was converted to land for residents and infrastructure land, and 29.18 km^2^ was converted to lakes. The increase in the lake area was primarily due to the implementation of water replenishment for the ecosystem measures. According to the statistics, during 1981–2019, emergency water replenishment was conducted 46 times in Baiyangdian Lake [43].

As can be seen from Table S3 in the Supplementary Materials, the conversion area from non-wetland to wetland was 28.44 km^2^ during 1980–2017, with a conversion rate of 1.93%. This was primarily represented by the conversion of dry land to rivers, lakes, ponds, and beach land. The conversion area from wetland to non-wetland was 111.72 km^2^, with a conversion rate of 37.74%. This primarily manifested in the conversion of beach land to paddy fields, dry land, and land for rural residents. Conversion in the internal wetland system was primarily manifested in the conversion of beach land to rivers, lakes, and ponds at conversion rates of 1.77%, 19.45%, and 2.74%, respectively.

### 3.2. Analysis of the Exponential Change in the Landscape Pattern

#### 3.2.1. Spatial Granularity Effect Analysis

A landscape pattern analysis has obvious scale effects that are generally expressed in terms of the spatial amplitude and spatial granularity. The spatial granularity effect is mainly due to the fact that in gridded maps, the edges of the tiles are always larger than the actual edges, and the shape of the edges will also be different. Therefore, the grid version will produce errors when calculating the edge parameters. This error depends on the resolution of the grid. Hence, the changes in the landscape pattern index under different granularity conditions need to be analyzed to determine the optimal granularity interval for analysis and select the best granularity for further analysis. The following six landscape-level indexes were used to select the optimal granularity of the landscape pattern index calculation from the influence of the scale on its own value and the influence on the change trend of the multi-year series.

**Figure 5:**
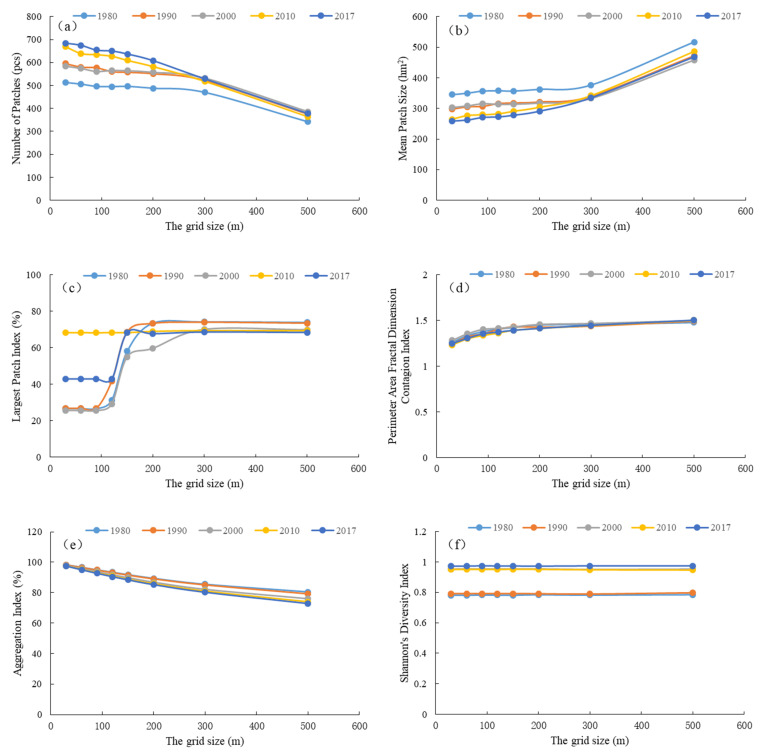
The relationship between the landscape pattern indexes of the Xiong’an New Area and its grid size. (**a**) NP, (**b**) MPS, (**c**) LPI, (**d**) PAFRAC, (**e**) AI, and (**f**) SHDI.

Figure 5 shows that the patch density, mean patch area, and largest patch index were more sensitive to changes in particle grid size, while the perimeter area fractal dimension, aggregation index, and Shannon’s evenness index were less sensitive to changes in the particle grid size. The first scale domain where the landscape index changes with the grid size can be used to determine the grid size, and the appropriate value range of the granularity of the landscape pattern can be analyzed, which is typically determined by the first scale turning point [44]. When selecting the granularity, it should not only ensure the quality of the calculation and reflect the overall characteristics of the landscape, but also not make the workload in the calculation process too large. Therefore, the medium-to-large granularity in the first scale domain was selected as the appropriate granularity. The above figure shows that the first scale domain was primarily concentrated from 30–120 m, and the multi-mutation effect of the largest patch index was obvious when the particle size was 120 m. In addition, it shows that the spatial granularity had little effect on the evolution trend of the landscape pattern index in the multi-year series of the Xiong’an New Area, and the change trend of the landscape pattern index in different years was basically the same. Because the area of the Xiong’an New Area is small, the amount of calculation was small, and there are small-scale ponds and other landscapes in the range. In order to avoid the loss of spatial information of small landscapes (such as patches), 30 m was used as the optimal granularity of the landscape in the Xiong’an New Area.

#### 3.2.2. Analysis of Class Scale Wetland Landscape Pattern

**Figure 6:**
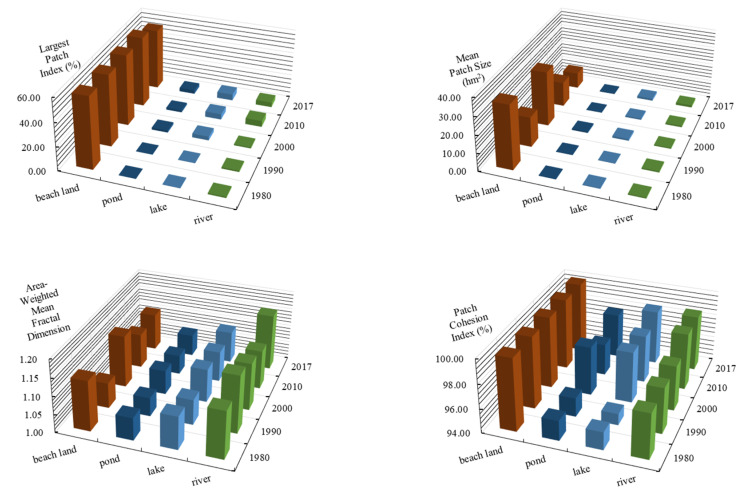
Changes in the landscape indexes (LPI, MPS, FRAC_AM, and COHESION) at the class scale in the Xiong’an New Area from 1980 to 2017.

The index analysis of the landscape pattern at the class scale was conducted for four wetland types: beach land, pond, lake, and river. The result is shown in Figure 6. The LPI is a simple method used to describe the degree of landscape dominance. After a comparison, it was found that the LPI of beach land significantly exceeded the other types, indicating that beach land was the largest wetland-dominant landscape type in the Xiong’an New Area. However, the landscape dominance of beach land showed a slight downward trend, and the landscape dominance of other wetland types also slightly increased. The MPS of the beach land was the largest among the wetland landscape types, but in recent years, it has decreased from 35.61 km^2^ in 1980 to 8.82 km^2^ in 2017. This result indicates that the beach land patches tended to be scattered. The PAFRAC of each wetland type was in the range of 1.00–1.20, with little change. The shape and complexity of the landscape patches were similar. Among them, the shape of the ponds was the least complex, and the shape of the rivers was the most complex. Prior to 2000, the combination of ponds and lakes was relatively small, indicating a greater degree of fragmentation. After 2000, the combination of ponds and lakes increased, and the degree of fragmentation decreased. When the plaque fragmentation of the ponds and lakes decreased, it indicated that there were fewer patches in small areas, but increased patches in large areas. In addition, the plaque connectivity was enhanced, so the fluidity of material, energy, and information was enhanced. This had a positive effect on the growth and reproduction of animals and plants living in ponds and lakes. This was primarily due to the strengthening of water replenishment for the ecosystem during the later period, and also demonstrated the benefits of water replenishment for the ecosystem.

#### 3.2.3. Landscape-Scale Wetland Landscape Pattern Analysis

**Figure 7:**
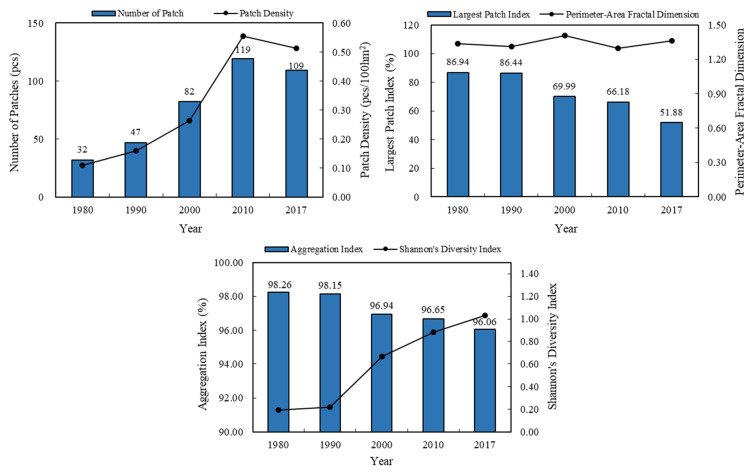
Changes in the landscape indexes (NP, PD, LPI, PAFRAC, AI, and SHDI) at the landscape scale in the Xiong’an New Area from 1980 to 2017.

The greater the number of patches and the greater the patch density, the higher the fragmentation of the landscape. Figure 7 shows that during 1980–2017, the number of wetland patches and the patch density showed a gradual increase. Compared with 1980, the number of patches increased from 32 to 109 in 2017 and the increase rate reached 20 per 10 years. The patch density rose from 0.11 to 0.51, and the increase rate reached 0.1 per 10 years. The increase in the number of patches and patch density indicated that the fragmentation of the wetland landscapes is increased. However, it can also be noted that from 2010–2017, both the number of patches and patch density have decreased, primarily due to the implementation and protection of ecological environment protection policies. The LPI reached a maximum value of 86.94 in 1980 and then began to decline, indicating that the landscape types began to become more uniform. The PAFRAC did not change much, fluctuating near 1.30, indicating that the patch shape was not too complicated. The degree of patch aggregation showed a decreasing trend from 1980 to 2017, indicating that the connectivity of similar landscapes had gradually decreased. The SHDI showed an increasing trend, indicating that the proportion of various landscape types had been balanced and landscape heterogeneity increased. Because differences in the landscape classification system will cause differences in the resulting landscape pattern index, the above analysis results are only applicable to the wetland landscape classification system used in this study.

### 3.3. Driving Factor Analysis

#### 3.3.1. Precipitation and Temperature

**Figure 8:**
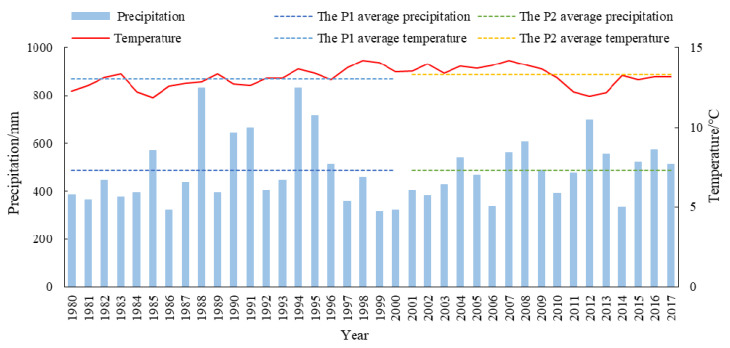
Changes in the precipitation and annual mean temperature in the Xiong’an New Area from 1980 to 2017. The data source: http://data.cma.cn/ (accessed on 10 October 2020).

As can be seen from the above analysis results, 2000 was a key time point for wetland changes in the Xiong’an New Area, so the driving factors were analyzed during different periods of time: 1980–2000 was used as the P1 period, and 2000–2017 was used as the P2 period. Precipitation and temperature are the primary meteorological factors that affect the wetland landscape pattern. Precipitation is an important water supply for wetlands. Increases in temperature directly affect evapotranspiration from the water’s surface and vegetation, thereby affecting the area of the wetland. Figure 8 shows changes in precipitation and annual mean temperature in the Xiong’an New Area from 1980 to 2017. It can be seen that P2 had fewer years with rich precipitation than P1, but the average annual precipitation was basically the same, indicating that the local precipitation in the Xiong’an New Area had little impact on wetland change. The annual average temperature in the P2 time period was greater than that in the P1 time period, indicating that the evaporation during this period increased, which would have reduced the wetland area to some extent.

#### 3.3.2. Hydrological Processes

**Figure 9:**
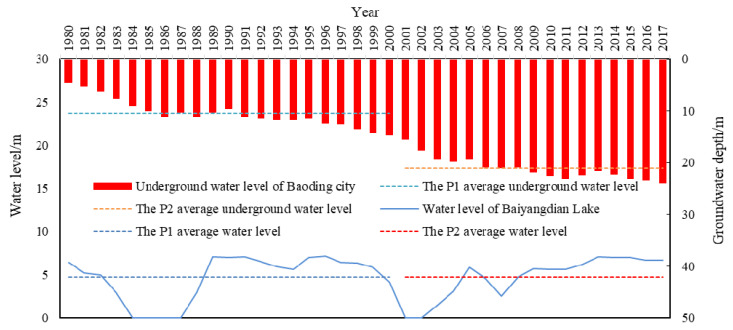
Changes in the groundwater level and Baiyangdian Lake water level in Baoding City from 1980 to 2017 [34,40].

**Figure 10:**
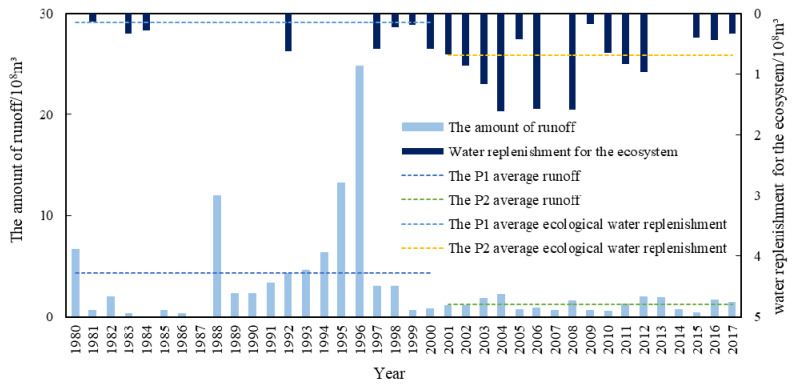
Changes in the water inflow into Baiyang Lake and the water replenishment for the ecosystem from 1980 to 2017 [35,36,37,38,39].

Hydrological conditions depend primarily on hydrological cycles and the conversion of natural energy. The change in the natural water level rhythm is an important manifestation of wetland hydrological processes that first has an effect on the physical and chemical environment of wetlands, and then affects the biological components of the ecosystem. The variation in the groundwater depth and surface water level in Baiyangdian Lake is an important factor that affects the landscape pattern of this area. Figure 9 shows the changes in the groundwater level and the Baiyangdian Lake water level in Baoding City from 1980 to 2017. It can be seen that the groundwater depth in the P1 time period was only 10.48 m, while that in the P2 time period was 21.07 m. This result indicated an obvious trend of increasing underground depth and serious groundwater exploitation. This can represent the change in the Baiyangdian groundwater level to some extent. Under these conditions, Baiyangdian Lake cannot be recharged by groundwater. The average annual water level of Baiyangdian Lake during the P1 and P2 periods showed little difference. However, in the 1980s and early 2000s, Baiyangdian Lake was dry for a few consecutive years, which caused great damage to the wetland ecosystem. Figure 10 shows the changes in the water inflow into Baiyang Lake and the water replenishment for the ecosystem from 1980 to 2017. It can be seen that the average annual natural runoff decreased significantly near 2000, from 434 million m^3^ during the P1 period to 122 million m^3^ during the P2 period.

**Figure 11:**
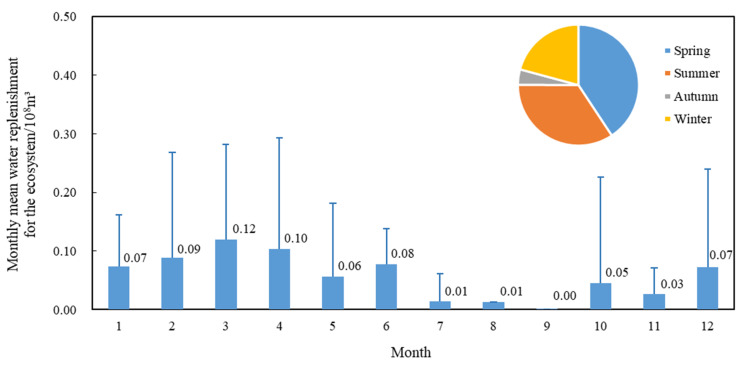
Monthly mean distribution corresponding to 38 years of water replenishment for the ecosystem in Baiyangdian Lake from 1980 to 2017 [35,36,37,38,39].

To alleviate the ecological crisis caused by water shortage, water replenishment for the ecosystem has been continuously strengthened, and the average annual water replenishment for the ecosystem during the P2 period reached 68 million m^3^. Water replenishment for the ecosystem measures can temporarily alleviate a water crisis in Baiyangdian Lake and keep the low water level running, but this cannot fundamentally solve the problem. When performing water replenishment for the ecosystem, it is also necessary to pay attention to the changes in the annual hydrological rhythm of Baiyangdian Lake. The needs of the Baiyangdian animal and plant habitats at the water level must also be considered. The ecological replenishment process should be adjusted to meet the needs of the Baiyangdian hydrological rhythm changes. Over the years, water replenishment for the ecosystem has primarily been concentrated from January to June (Figure 11), and the water replenishment for the ecosystem amount has been the largest during March and April. However, according to the historical water level change process, the demand for water volume has been greater from August to October, while the water replenishment for the ecosystem volume during this period has been less.

#### 3.3.3. Socioeconomic Factors

**Figure 12:**
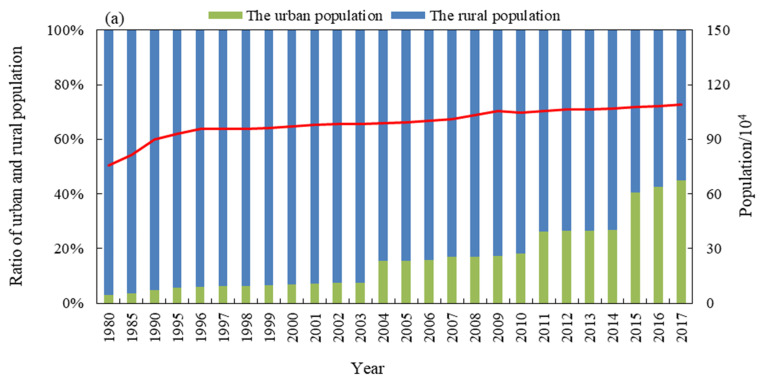

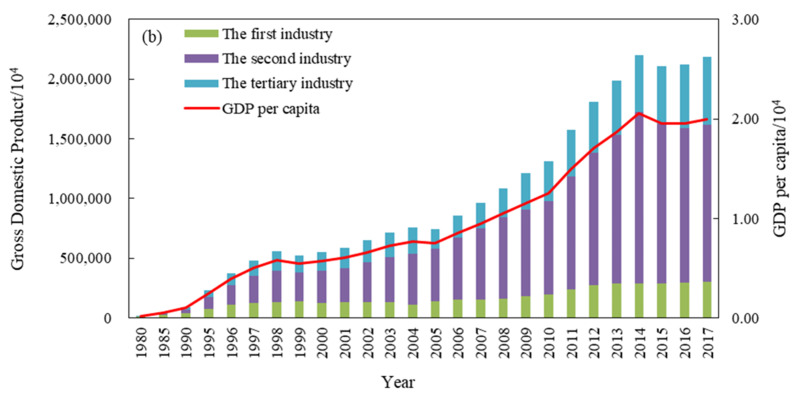
Changes in the economic and social factors in the Xiong’an New Area from 1980 to 2017. (**a**) Population and (**b**) economy. The data source: Baoding City Economic Statistics Yearbook (https://data.cnki.net/area/Yearbook/Single/N2017120334?z=D03 (accessed on 20 November 2020)).

Regional population and economic development level are important indicators that can be used to represent the degree to which human activities interfere with the ecological environment. From the perspective of population change (Figure 12a), the population growth rate was relatively fast prior to 2000, but relatively slow after 2000. The change in the proportion of the urban population and rural population shows that the proportion of urban population has been increasing, and the trend has significantly expanded since 2000. These two trends will increase the domestic water consumption in the region. Combined with the significant increase in the GDP (Figure 12b), this indicates that the increase in industrial and agricultural water consumption will reduce the amount of water flowing into Baiyangdian Lake, thus reducing the area of the wetlands.

#### 3.3.4. Principal Component Analysis

SPSS was used to perform the PCA on the driving force factors of Baiyangdian Lake, and the correlation matrix of the driving force factors for wetland changes was obtained. The results showed that there was a strong correlation between the factors affecting the area changes of Baiyangdian Lake. For example: X_3_ and X_7_–X_12_, X_7_ and X_8_–X_13_, X_9_ and X_10_–X_11_, X_10_ and X_11_–X_13_, X_11_ and X_12_–X_13_, and other correlations were greater than 0.850. If these factors were directly used to analyze the wetland area and landscape changes in the driving mechanism, a large portion of the information would overlap, which would increase the difficulty of analysis. Therefore, it was necessary to conduct a PCA of the driving factors and select several principal components that contained most of the information.

The results of the characteristic value and contribution rate of each principal component showed that the first principal component explained 58.21% of the total variables, the second principal component explained 16.15% of the total variable, and the third principal component explained 10.92% of the total variable. The principal component loading reflected the correlation between the original variable and the principal component. It represents the weight of each original variable in the principal component. The greater the absolute value of the load, the closer the relationship between the corresponding variable and the principal component. The analysis found that in the initial principal component load matrix, the difference between the factors was not obvious, and the load reflected too much information. To better explain the principal component, the method of maximum variance rotation was used to rotate the initial load matrix to obtain the rotated principal component load matrix.

The end result was that X_11_, X_7_, X_8_, X_10_, X_9_, X_12_, X_13_, and X_3_ had larger loads in the first principal component. These factors all reflected economic and social development. Among them, X_3_ was directly affected by artificial groundwater withdrawal. Therefore, the first principal component could be considered as a representative of economic and social development. X_2_, X_14_, and X_1_ had larger loads in the second principal component. These factors primarily reflected the influence of meteorological conditions. X_4_, X_5_, and X_6_ had a large load on the third principal component. These factors primary reflect the influence of hydrological processes. The above analysis showed that the leading factors of the wetland landscape changes in the Xiong’an New Area can be summarized as socio-economic development and meteorological and hydrological processes, of which socio-economic development was the most important factor. Please refer to the Supplementary Materials (Tables S4–S6) for the detailed calculations of the data above.

### 3.4. Uncertainty Analysis

In the interpretation of land use landscape types, attention should be paid to selecting remote sensing images at an appropriate time. The improper selection of images directly affects the authenticity of the interpretation results. When studying the evolution of land use landscape patterns over many years, the year in which a sudden change occurred in the areas of the dominant landscape types should be considered as much as possible. For Baiyangdian, the phenomenon of dry lakes that occurred in the years after 1980 and the beginning of the 20th century should be considered. This study primarily considered the similar selections of 1980 and 2000. Relatively speaking, the analysis results of the mutation characteristics will be slightly lacking, but this had little effect on the overall results. The month selected of the remote sensing images also has a great impact on the analysis results, which is primarily due to changes in the water level during the year and the phenological characteristics of the aquatic plants [31]. To better identify wetland and other landscape types, the seasons for growing aquatic plants and crops should be selected based on the influence of cloud cover. These seasons, combined with changes in the water level process of the Baiyangdian Lake during the year, suggest that remote sensing images from July and August should be selected. When the landscape pattern index was used to analyze the land use landscape pattern, the uncertainty in the landscape pattern index analysis method can be transmitted and accumulated in different analysis stages [45]. Therefore, in practical applications, on the basis of understanding the actual meaning of the landscape pattern index, careful selection and interpretation should be combined with ecological processes [46].

## 4. Conclusions

The wetland ecosystem in the Xiong’an New Area is rich and diverse, but it is also very fragile due to the characteristics of the ecosystem type and the surrounding environment. As an important part of the wetland ecosystem in the Xiong’an New Area, Baiyangdian Lake is also the largest shallow water lake in the North China Plain, which is greatly affected by human activities.

The area of wetland in the Xiong’an New Area has decreased significantly since 2000. As the most dominant wetland landscape, beach land has also declined in recent years and has been gradually occupied by cultivated and construction land. Since 2000, the area of lakes, rivers, and pond wetlands has increased due to ecological rehydration. Both the number of wetland patches and the patch density have shown a gradual increasing trend, and the increasing rates have reached 20 per 10 years and 0.1 per 10 years, respectively. The connectivity of similar landscapes has gradually decreased, but the Shannon diversity index showed an increasing trend, indicating that the proportion of landscape types tended to be balanced and the heterogeneity of the landscapes increased. The results of the driving analysis showed that an increase in temperature will aggravate the decrease in wetland area to a certain extent, and water replenishment for the ecosystem can alleviate the ecological crisis caused by water shortage. However, it will not be enough to bring about fundamental changes. Moreover, attention should be paid to the reasonable distribution of annual processes during water replenishment for the ecosystem. An increase in the population and the development of industry and agriculture have increased the amount of water used in the region, and the natural runoff flowing into the wetland system has decreased sharply. This is the primary reason for wetland degradation in the Xiong’an New Area. PCA showed that social and economic factors are the primary driving factors that are affecting the wetland landscape changes in the Xiong’an New Area.

Emphasis on wetland research, management, protection, and ecology to achieve sustainable wetland development is essential to protect the environment. In the early stage of economic and social development, people may be trapped in immediate short-term interests and ignore wetland protection. However, with the awakening of environmental protection consciousness, people have begun to coordinate the relationship between social and economic development and environmental protection to achieve the maximum of comprehensive benefits. The results of this study will help to improve the analysis system for wetland ecosystem evolution and provide a reference for the establishment of an effective mechanism of wetland conservation. Future work should be done regarding the development of a reasonable prediction model and the optimal allocation of the wetland landscape pattern.

## Figures and Tables

**Table 1:** Landscape type classification system of the Xiong’an New Area.

Landscape Type	Major Type	Minor Type
Wetland	Water body	River
		Lake
		Pond
		Beach land
Non-wetland	Arable land	Paddy field
		Dry land
	Woodland	Other woodland
	Grassland	High coverage grassland
		Low coverage grassland
	Construction land	Land for urban residents
		Land for rural residents
		Infrastructure land

**Table 2:** Indexes used in the study and their ecological meaning in the landscape pattern.

Index	Expressions	Unit	Applied Scale	Ecological Meaning
largest patch index (LPI)	$\mathrm{LPI}=\frac{Max\left(a_{1},\ldots ,a_{n}\right)}{A_{i}}\times 100$	%	Patch class/landscape	Describe the dominant landscape
mean patch area (MPS)	$\mathrm{MPS}=\frac{1}{n}\sum_{j=1}^{n}a_{ij}$	hm^2^	Patch class	Describing the degree of fragmentation
area weighted mean fractal dimension (FRAC_AM)	$\mathrm{FRAC}\_\mathrm{AM}=\sum_{i=1}^{m}\left[\frac{2\ln\left(0.25P_{i}\right)}{ln\left(a_{i}\right)}\left(\frac{a_{i}}{A_{i}}\right)\right]$	None	Patch class	Describing the degree of complexity of the patch shapes
patch cohesion index (COHESION)	$\mathrm{COHESION}=\left[1-\frac{\sum_{i=1}^{m}\sum_{j=1}^{n}P_{ij}}{\sum_{i=1}^{m}\sum_{j=1}^{n}P_{ij}\sqrt{a_{ij}}}\right]{\left[1-\frac{1}{\sqrt{A_{i}}}\right]}^{-1}\times 100$	%	Patch class	Describe the connectivity of plaques
number of patches (NP)	NP = *N*	Pcs	landscape	Describe the total number of landscapes
patch density (PD)	PD = *N/A*	Pcs/hm^2^	landscape	Describe the degree of landscape fragmentation
perimeter area fractal dimension (PAFRAC)	$\mathrm{PAFRAC}\;=\frac{2\times \left\{\left[N\sum_{1}^{m}\sum_{1}^{n}lnP_{ij}^{2}\right]-\left[\sum_{1}^{m}\sum_{1}^{n}lnP_{ij}^{}\right]\right\}}{\left[N\sum_{1}^{m}\sum_{1}^{n}\left(lnP_{ij}-lna_{ij}\right)\right]-\left[\left(\sum_{1}^{m}\sum_{1}^{n}lnP_{ij}^{}\right)\left(\sum_{1}^{m}\sum_{1}^{n}lna_{ij}^{}\right)\right]}$	None	landscape	Describe the complexity of landscape shape
aggregation index (AI)	$\mathrm{AI}=\left[\sum_{i=1}^{m}\left(\frac{g_{ii}}{\max g_{ii}}\right)p_{i}\right]\times 100$	%	landscape	Describe the degree of landscape aggregation
Shannon’s diversity index (SHDI)	$\mathrm{SHDI}=-\sum_{i=1}^{m}p_{i}\left(lnp_{i}\right)$	None	landscape	Describing landscape diversity

In the table, *i* = 1, …, *m*, is the patch class, *j* = 1, …, *n*, is the patch number; *m* is the total number of the landscape; *n* is the total number of patch class; *a* represents patch area; *A* represents landscape area; *P* represents plaque perimeter; *N* represents the total number of plaques; *g* represents the number of pixels between different patches of the same patch type; *p* represents the proportion of landscape area occupied by each patch type.

## Data Availability

The data presented in this study are available on request from the corresponding author.

## Supplementary Materials

The following are available online at https://www.mdpi.com/article/10.3390/ijerph18094403/s1, Table S1: Landscape Transfer Matrix of the Xiong’an New Area from 1980 to 2000, Table S2: Landscape Transfer Matrix of the Xiong’an New Area from 2000 to 2017, Table S3: Landscape Transfer Matrix of the Xiong’an New Area from 1980 to 2017, Table S4: Correlation matrix of the driving factors of wetland change, Table S5: Eigenvalues and principal component contribution rates, Table S6: Principal component load matrix after rotation.
