# Supplementary material for: Landscape Pattern Evolution Processes of Wetlands and Their Driving Factors in the Xiong’an New Area of China
Source: Int J Environ Res Public Health. 2021 Apr 21;18(9):4403. doi: 10.3390/ijerph18094403 (PMC8122455; doi:10.3390/ijerph18094403)
Supplement: Supplementary file 1 [file ijerph-18-04403-s001.zip › ijerph-1130270-supplementary.pdf]

Table S1. Landscape Transfer Matrix of the Xiong'an New Area from 1980 to 2000 (unit: km²).

1980 \ 2000	Dry land	Other woodland	Low coverage grassland	High coverage grassland	River	Lake	Pond	Beach land	Land for urban residents	Land for rural residents	Infrastructure land	Sum
**Dry land**	1241.44	6.37				6.94	0.54	10.54	9.32	37.75	2.60	1315.50
**Other woodland**	0.07	4.43										4.50
**Low coverage grassland**			0.36									0.36
**High coverage grassland**				0.16								0.16
**River**					6.91							6.91
**Lake**						2.78		0.10				2.88
**Pond**							1.34					1.34
**Beach land**	1.83					40.33	7.17	235.13		0.44		284.90
**Land for urban residents**									7.81			7.81
**Land for rural residents**									1.72	137.36		139.08
**Infrastructure land**									0.42		5.70	6.12
**Sum**	1243.34	10.81	0.36	0.16	6.91	50.05	9.05	245.77	19.26	175.55	8.30	1769.57

Table S2. Landscape Transfer Matrix of the Xiong'an New Area from 2000 to 2017 (unit: km^2).

2000 \ 2017	Paddy field	Dry land	Other woodland	River	Lake	Pond	Beach land	Land for urban residents	Land for rural residents	Infrastructure land	Sum
**Dry land**	58.23	1000.21	5.04	8.13	0.83	2.15	6.35	12.49	129.08	20.82	1243.34
**Other woodland**	0.36	4.94	3.65	0.00				0.13	1.57	0.15	10.81
**Low coverage grassland**	0.14								0.22		0.36
**High coverage grassland**		0.09								0.06	0.16
**River**		3.98	0.09	2.46				0.00	0.38		6.91
**Lake**		0.55		0.96	30.37	0.00	16.56		1.28	0.34	50.05
**Pond**		1.17		0.17	0.48	5.01	1.72		0.50	0.01	9.05
**Beach land**	13.97	85.35	0.03	4.01	29.18	3.33	97.08	0.36	11.76	0.71	245.77
**Land for urban residents**		1.95						17.31	0.00		19.26
**Land for rural residents**	1.54	44.51	0.51	0.75	0.39	0.44	1.73	1.63	119.67	4.37	175.55
**Infrastructure land**	0.03	4.92		0.12	0.03	0.47	0.06	0.11	0.16	2.41	8.30
**Sum**	74.27	1147.68	9.31	16.61	61.27	11.39	123.49	32.03	264.63	28.89	1769.57

Table S3. Landscape Transfer Matrix of the Xiong'an New Area from 1980 to 2017 (unit: km^2).

1980 \ 2017	Paddy field	Dry land	Other woodland	River	Lake	Pond	Beach land	Land for urban residents	Land for rural residents	Infrastructure land	Sum
**Dry land**	59.37	1025.41	8.02	8.28	4.34	2.41	10.20	20.82	154.10	22.54	1315.50
**Other woodland**	0.36	2.38	0.81						0.80	0.15	4.50
**Low coverage grassland**	0.14								0.22		0.36
**High coverage grassland**		0.09								0.06	0.16
**River**		3.98	0.09	2.46				0.00	0.38		6.91
**Lake**		0.11			1.14		1.34		0.29		2.88
**Pond**		0.28		0.10	0.00	0.56	0.03		0.37		1.34
**Beach land**	12.87	78.93	0.03	5.03	55.41	7.80	110.45	0.36	13.00	1.03	284.90
**Land for urban residents**		0.14						7.67			7.81
**Land for rural residents**	1.53	33.02	0.37	0.73	0.35	0.44	1.44	2.66	95.31	3.22	139.08
**Infrastructure land**		3.33			0.03	0.19	0.02	0.51	0.16	1.88	6.12
**Sum**	74.27	1147.68	9.31	16.61	61.27	11.39	123.49	32.03	264.63	28.89	1769.57

Table S4. Correlation matrix of the driving factors of wetland change

	X_1	X_2	X_3	X_4	X_5	X_6	X_7	X_8	X_9	X_{10}	X_{11}	X_{12}	X_{13}	X_{14}
X_1	1	-0.355	0.064	0.178	0.237	-0.1	0.106	0.059	0.119	0.094	0.094	0.073	0.219	-0.27
X_2	-0.355	1	0.114	-0.177	-0.113	0.271	-0.174	-0.109	-0.192	-0.152	-0.154	0.172	-0.192	0.449
X_3	0.064	0.114	1	0.074	-0.424	0.388	0.863	0.859	0.854	0.868	0.874	0.942	0.797	-0.38
X_4	0.178	-0.177	0.074	1	0.298	-0.344	0.353	0.36	0.36	0.322	0.344	0.244	0.377	-0.36
X_5	0.237	-0.113	-0.424	0.298	1	-0.285	-0.329	-0.29	-0.334	-0.326	-0.335	-0.285	-0.285	0.181
X_6	-0.1	0.271	0.388	-0.344	-0.285	1	0.078	0.088	0.065	0.109	0.096	0.27	0.059	0.151
X_7	0.106	-0.174	0.863	0.353	-0.329	0.078	1	0.979	0.998	0.991	0.999	0.87	0.942	-0.668
X_8	0.059	-0.109	0.859	0.36	-0.29	0.088	0.979	1	0.966	0.978	0.982	0.909	0.903	-0.573
X_9	0.119	-0.192	0.854	0.36	-0.334	0.065	0.998	0.966	1	0.98	0.996	0.85	0.94	-0.681
X_{10}	0.094	-0.152	0.868	0.322	-0.326	0.109	0.991	0.978	0.98	1	0.99	0.881	0.945	-0.661
X_{11}	0.094	-0.154	0.874	0.344	-0.335	0.096	0.999	0.982	0.996	0.99	1	0.881	0.935	-0.647
X_{12}	0.073	0.172	0.942	0.244	-0.285	0.27	0.87	0.909	0.85	0.881	0.881	1	0.784	-0.309
X_{13}	0.219	-0.192	0.797	0.377	-0.285	0.059	0.942	0.903	0.94	0.945	0.935	0.784	1	-0.833
X_{14}	-0.27	0.449	-0.38	-0.36	0.181	0.151	-0.668	-0.573	-0.681	-0.661	-0.647	-0.309	-0.833	1

Table S5. Eigenvalues and principal component contribution rates

The principal components	The eigenvalue	contribution /%	Cumulative contribution rate/%	The principal components	The eigenvalue	contribution /%	Cumulative contribution rate/%
1	8.149	58.21	58.21	8	0.068	0.484	99.555
2	2.26	16.145	74.354	9	0.042	0.303	99.858
3	1.11	10.929	85.284	10	0.012	0.085	99.943
4	0.965	3.894	89.178	11	0.008	0.057	99.999
5	0.536	3.826	93.003	12	9.85E-05	0.001	100
6	0.477	3.407	96.41	13	1.61E-09	1.15E-08	100
7	0.372	2.661	99.071	14	7.82E-17	5.58E-16	100

Table S6. Principal component load matrix after rotation

Variable	The first principal component	The second principal component	The third principal component	Variable	The first principal component	The second principal component	The third principal component
X_{11}	0.983	-0.141	-0.032	X_3	0.908	0.127	-0.28
X_7	0.979	-0.168	-0.022	X_2	-0.007	0.875	-0.074
X_8	0.979	-0.062	0.017	X_{14}	-0.586	0.655	-0.044
X_{10}	0.978	-0.147	-0.044	X_1	0.058	-0.561	0.173
X_9	0.97	-0.193	-0.021	X_4	0.377	-0.11	0.78
X_{12}	0.935	0.223	-0.054	X_5	-0.319	-0.001	0.735
X_{13}	0.927	-0.297	-0.003	X_6	0.15	0.33	-0.632
